# The Connection of Dental with Medical Education

**Published:** 1882-07

**Authors:** 


### EDITORIAL, ETC.

---

*The Connection of Dental with Medical Education*—When the Editor of this Journal entered the dental profession in the year 1855, the highest aim of the most reputable dental practitioners, and especially of those directly connected with dental teaching, was the recognition of dental science as a department or specialty of medicine. The organization of Dental in connection with Medical Departments by such old and honored Universities of Medicine as Pennsylvania, Harvard and Maryland, now established facts, would at that day, as it has at the present, have elicited the warmest commendations, and been received as an evidence of the appreciation of the dental art, not only by the medical profession, but by the intelligent portion of the public. This desire for recognition, had not, during the many years it remained ungratified, become extinct, and every respectable practitioner of dentistry who has the honor of his profession at heart, must welcome such an appreciation of the dental art, and be fully convinced of its importance in elevating the status of dentistry to an equality with the profession of the occulist and aurist.

Although the subject of dental education had engaged the attention of some of the most talented members of the medical profession for many years, yet until within a few years past, they were unwilling to accord to it a rank equal to that of other reccgnized specialties in their older and honored science. The principal reason for such opposition in the past, was the comparatively small number of educated dental practitioners; the majority of those who professed to practice dentistry being persons without any professional education or knowledge beyond mere handicraft, and who had forsaken various mechanical pursuits to engage in the practice of an art which they considered to be more remunerative and less laborious. And while the desire for recognition on the part of the mother science has been cherished up to the present time, it has only been within the last two decades that dental science has become worthy of being ranked as a department of medicine. Within the time referred to a greater number of educated gentlemen have adopted dentistry as a profession, and by their scientific investigations and valuable inventions, have accomplished the truly wonderful and important improvements which have had such an influence in placing it in the position it now occupies, and rendering it worthy of the recognition now accorded it.

It was the dearest wish of the principal founder of the oldest of our dental colleges, as expressed to the writer on more than one occasion, to have the institution, in whose welfare he was so deeply interested, united with the Medical Department of the University of Maryland. And we feel perfectly safe in stating, that if the majority of the dental practitioners now connected with separate dental schools, could secure similar positions in Dental Departments connected with Medical Universities, such dental schools would cease to exist, and dental teaching be conducted where superior advantages are presented to the dental student. The intelligent public will soon begin to discriminate between the young men who are educated in a manner equal to the demands of their science, and those who are taught the mere handicraft of their art, and whose ability consists in manipulative skill, without the knowledge required to properly direct such skill. The result will be a higher grade of dental graduates, who can justly claim an *esprit de corps* which will serve as a stimulus to both study and practice. That the days are numbered of such dental schools as are in the habit of advising their students that the Infirmary and Laboratory will furnish all the knowledge required for the practice of dentistry and that Chairs of Clinical Dentistry are unnecessary. is beyond doubt. When practical instruction wholly devolves upon demonstrators, such a course must surely result in a limited experience, and prove detrimental to the student who is compelled to confine himself to a particular mode of practice. which may not in all cases be a reliable one, or to the use of a particular material to the exclusion of others equally useful.

The science of dentistry in its present status requires more than mere manipulative skill; its practitioners must be properly educated in its theory also, for it must be remembered that the reputation of every profession depends upon the intelligence of its practitioners.

We should educate our dental students in such a manner as will enable them to meet the family physician in consultation, when the nature of a case requires it, and intelligently furnish the dental details on which the efficacy of his constitutional treatment may depend. The dental students should also, in many cases, be able to prescribe such constitutional treatment if called upon to do so; and such occasions are by no means rare in a dental practice. And even if the patient, before resorting to the prescription of the dentist, should consult his physician, the intelligent opinion of the former cannot fail to prove beneficial by an increase of respect for, and greater confidence in his professional ability on the part of the patient.

Everyone judging impartially, must admit that the advantages offered to dental students by Dental Departments in Medical Universities of well established reputation, will enable such students when they become practitioners of dentistry to better meet the requirements of their profession, and enjoy to a higher degree the confidence of the community in which they locate. Taking the Dental Department of the University of Maryland for an example, let us briefly refer to the connection existing between its medical and dental departments. In the first place, the Dental Department of the University of Maryland forms an integral part of that old and honored institution which was organized in the year 1806, by Nathaniel Potter, M, D., John B. Davidge M. D., Jas. Cocke, M. D., Thomas E. Bond, M. D., Wm. Donaldson, M. D., and others, and which has always maintained an enviable reputation throughout the entire country. Its Dental Department is not a mere appendage nor is the connection so established a pretended one made merely as an offset to other institutions which have followed a like course.

On the contrary, the dental student in the University of Maryland, has all the advantages of the medical student in addition to the dental instruction he receives from the dental practitioners who compose a portion of its faculty, and who have been connected with dental teaching in another institution for very many years. The superior advantages to the dental student of an extensive University of Medicine cannot be overestimated, whether such student desires to confine himself to the study of dental science alone, or intends to become a graduate of medicine also. The opportunities for aquiring knowledge from the Surgical Clinics in a Hospital which receives 24,000 patients annually, and the experience to be gained from the Oral Surgery Clinics by an accomplished Surgeon whose reputation is so well established, should certainly prove incentives and enable the dental student to meet all the demands upon his professional skill which after practice may present.

It is beyond question the fact that the proper place for the study of dentistry is the Dental Department of a reputable Medical University when the connection is so intimate and veritable as that which exists between the Medical and Dental Departments of the University of Maryland. It should also be remembered that the dental diplomas of the University of Maryland Dental Department will bear a date *thirty-four years older* than those of any other *dental school.*

